# A cross‐sectional study of community‐level physician retention and hospitalization in rural Ontario, Canada

**DOI:** 10.1111/jrh.12661

**Published:** 2022-03-15

**Authors:** Maria Mathews, Alexandra M. Ouédraogo, Melody Lam, Peter Gozdyra, Michael Green

**Affiliations:** ^1^ Department of Family Medicine Western University Schulich School of Medicine & Dentistry, Western Centre for Public Health and Family Medicine London Ontario Canada; ^2^ ICES Western ICES London Ontario Canada; ^3^ ICES Central ICES Toronto Ontario Canada; ^4^ Department of Family Medicine Queen's University Kingston Ontario Canada

**Keywords:** family physician, hospitalization, primary care, retention, rural

## Abstract

**Purpose:**

Many rural communities experience poor family physician retention. We examined the association between community‐level physician retention and hospitalization for all causes and ambulatory care‐sensitive conditions (ACSCs) in 2017 among residents of rural communities in Ontario, Canada.

**Methods:**

We conducted a population‐based cross‐sectional study by linking administrative data from the public health insurance program in Ontario. To create the physician retention measure for each rural community, we divided the number of family physicians who worked in the community in both 2016 and 2017 by the total number of unique family physicians in the community in either year. We grouped retention level by tertile and added a fourth category, “no physician” to include communities that did not have any residing physicians in either 2016 or 2017. Outcomes were all‐cause hospitalization and ACSC hospitalization between April 1, 2017 and March 31, 2018.

**Findings:**

Among 1,436,794 rural residents, there were 93,752 all‐cause hospitalizations and 8,691 ACSC hospitalizations in 2017. After controlling for other predictors, compared to residents in low‐retention communities, residents of medium‐ and high‐retention communities were 0.888 (95% CI: 0.868‐0.909) and 0.937 (95% CI: 0.915‐0.960) times as likely to have all‐cause hospitalization, and residents of high‐retention communities were 0.918 (95% CI: 0.858‐0.981) times as likely to have ACSC hospitalization in 2017.

**Conclusions:**

Community‐level physician retention is significantly associated with all cause and ACSC hospitalization in rural communities in Ontario, and may serve as an alternate measure to assess the impact of disrupted continuity of care.

Rural residents have poorer health outcomes, such as higher rates of hospitalization and preventable mortality, than urban residents.^1^, ^2^, ^3^ The lack of context‐relevant measures of health care quality hinders efforts to improve health care in rural communities. Many rural communities across Canada have a high turnover of family physicians, and patients from these communities describe repeatedly seeing different physicians who do not know them well.^4^, ^5^ Physician turnover disrupts continuity of care, and is believed to lead to poorer quality‐of‐care by eroding the trust between patients and family physicians, reducing opportunities for patients to receive preventative services, lowering the quality of communication and diminishing physicians’ understanding of the patient's medical and personal history.^6^, ^7^, ^8^, ^9^, ^10^, ^11^, ^12^

High continuity of care has been shown to improve the quality of primary health care.^11^, ^12^ Researchers have constructed a number of metrics to measure continuity of care using administrative fee‐for‐service billing data, including the Usual Provider Continuity Index and Concentration of Care Index.^12^, ^13^, ^14^, ^15^ However, in many rural communities in Canada, family physicians are paid by alternate payment plans and may not submit shadow billings. As a result, these traditional metrics of continuity of care cannot be used to research the quality of primary health care in rural communities.

Measuring physician retention offers a potential alternative.^10^ This study will examine the association between physician retention and hospitalization for all causes and ambulatory care‐sensitive conditions (ACSCs) over a 1‐year period among residents of rural communities in Ontario that are outside the commuting zones of larger urban centers, and with less than 10,000 population. Also known as preventable hospitalizations, ACSC hospitalizations are an indicator of poor primary health care and are defined as hospitalizations for any of the following 7 conditions: angina, asthma, congestive heart failure (CHF) and pulmonary edema, chronic obstructive pulmonary disease (COPD), diabetes, grand mal seizures, and other epileptic seizures and hypertension.^12^ All cause and ACSC hospitalization have been shown to vary with established measures of continuity of care.^12^, ^14^, ^15^ We hypothesize that residents of rural communities with low physician retention will have a greater likelihood of being hospitalized for all cause and ACSC hospitalization in a 1‐year period than residents of rural communities with high physician retention, but a lower likelihood of all cause and ACSC hospitalization than communities with no residing physician. This initial, exploratory study will assess whether community‐level physician retention serves as an alternate measure of access and continuity of care in rural communities.

## METHODS

We conducted a population‐based cross‐sectional study using linked health administrative data held at ICES, an independent, nonprofit research institute funded by an annual grant from the Ontario Ministry of Health and the Ministry of Long‐Term Care. As a prescribed entity under Ontario's privacy legislation, ICES is authorized to collect and use health care data for the purposes of health system analysis, evaluation, and decision support. Secure access to these data is governed by policies and procedures that are approved by the Information and Privacy Commissioner of Ontario. The use of the data in this project is authorized under section 45 of Ontario's Personal Health Information Protection Act and does not require review by a Research Ethics Board.

We linked demographic information on individuals covered by the Ontario Health Insurance Program (OHIP) (public, universal health insurance program for medically necessary physician and hospital health services), with physician billings, hospitalizations, emergency room visits, practicing physicians, medical practice organizations, and geographic data from the national census. These datasets were linked using unique encoded identifiers and analyzed at ICES.

We included individuals covered by OHIP as of April 1, 2017. We excluded individuals who had missing or invalid age or sex data; died on or before April 1, 2017; were not residents of Ontario; were missing postal codes, census subdivision, or Local Health Integration Network (LHIN) regional data; had no record of contact with the health system for at least 7 years and were not eligible for OHIP. Urban residents were then excluded to create a cohort of residents living in rural census subdivisions (ie, communities). Rural census subdivisions are small towns and rural communities outside the commuting zones of larger urban centers, and with less than 10,000 population. We included only communities with less than 5% of its population working in larger communities (based on Statistics Canada's metropolitan influenced zones measure)^16^ to limit the likelihood that rural residents were accessing care outside their home community. This definition of rural is recommended by Statistics Canada^16^ and has been used in other studies of rural health care in Canada.^3^, ^10^ Demographic data on individuals were derived from the Registered Persons Database (RPDB), which contains basic demographic information on all individuals covered by OHIP.

The main exposure was physician retention. To create the physician retention measure, we first identified all physicians who worked in a rural census subdivision in 2016 (between April 1, 2016 and March 31, 2017) and in 2017 (between April 1, 2017 and March 31, 2018). For each rural community, we divided the number of physicians who worked in the community in both 2016 and 2017 by the total number of unique physicians in the community in either year. We grouped retention level by tertile (where high retention was 83.34%‐100%, moderate retention was 66.68%‐83.33%, and low retention was 0.1%‐66.67%). The cutoffs were based on the number of communities in each group, and consensus among authors that these cutoffs were consistent with general experience (ie, 5 of 6 physicians remained in the same community from 1 year to the next was considered good retention). In addition, we created a fourth category, “no physician” to include communities that did not have any physician whose main address was in the community in either 2016 or 2017.

Outcomes were all‐cause hospitalization (yes/no) and ACSC hospitalization (yes/no) between April 1, 2017 and March 31, 2018. Hospitalizations were identified using the Discharge Abstract Database, which is compiled by the Canadian Institute for Health Information and contains administrative, clinical (diagnoses and procedures/interventions), demographic, and administrative information for all admissions to acute care hospitals in Ontario. ACSC hospitalizations were defined as admissions where International Statistical Classification of Diseases and Related Health Problems, Tenth Revision, Canada (ICD‐10‐CA) codes indicated that any of the 7 ACSC conditions was the most responsible diagnosis.

Covariates included patient, community, and health service variables. Patient‐level covariates were age, sex, and number of comorbidities; patient age and sex were identified in the RPDB. The number of comorbidities was based on the presence of any of asthma, CHF, COPD, diabetes, hypertension, dementia, human immunodeficiency virus (HIV), rheumatoid arthritis, or Crohn's/colitis. Individuals with these comorbidities were identified from databases (Ontario asthma database, CHF database, COPD database, Ontario diabetes dataset, Ontario hypertension dataset, Ontario HIV database, Ontario rheumatoid arthritis dataset, and Ontario Crohn's and colitis cohort dataset) of validated condition‐specific cohorts that were developed by ICES using hospitalization, physician billing, emergency department, and same day surgery data.^17^, ^18^, ^19^, ^20^, ^21^, ^22^, ^23^, ^24^, ^25^, ^26^, ^27^, ^28^, ^29^, ^30^, ^31^, ^32^ The community‐level variables were defined as physicians per 1,000 population in the community. Health service variables were the type of primary care organization to which the patient belonged, and number of primary care, specialist, and emergency department visits between April 1, 2017 and March 31, 2018. Primary care organization describes different practice and funding models introduced in Ontario as part of a series of primary care reforms.^33^, ^34^ Most physicians belong to organizations that are patient enrollment models and have a defined roster of patients. As part of enrollment with a physician, patients agree not to seek care from physicians outside the group for nonurgent conditions. Capitation (CAP)‐funded organizations are physician group practices without an allied health team. Family health teams (FHTs) are capitation‐funded physician group practices with allied health teams. Physicians paid by fee‐for‐service models can be in practice models that either requires patient enrollment (FFS) or do not (NOG). Other group practices (OGPs) include a number of alternate payment mechanisms used in rural and northern communities, including nurse practitioner‐led clinics. Patients in the no physician (NOP) group are unattached (have not rostered with a physician practice and/or do not have a doctor they regularly see).

All analyses were conducted using SAS version 9.4 (SAS Institute, Cary, NC). After describing the characteristics of the sample, we used ANOVA or chi‐square tests to compare the characteristics of residents who lived in communities with different levels of physician retention. We used Pearson *r* correlation and variable inflation factor tests to assess multicollinearity between variables a priori. We used univariable and multivariable logistic regression to examine the relationship between each predictor and each outcome. Multivariable regression adjusted for other significant covariates and age‐sex interaction, which was identified through the bivariate analyses. The final multivariate regression model includes only statistically significant predictors and interaction terms.

In sensitivity analyses, we excluded communities in the northernmost health regions (referred to as LHIN at the time of the study) and repeated our analyses. These regions of the province have a high proportion of Indigenous people who live in remote and isolated areas and have health services delivery and governance that differ from communities in the rest of the province. We also repeated our analysis of ACSC hospitalization to adults 18‐74 years, consistent with more restricted definitions of ACSC.^12^, ^14^

## RESULTS

**FIGURE 1:**
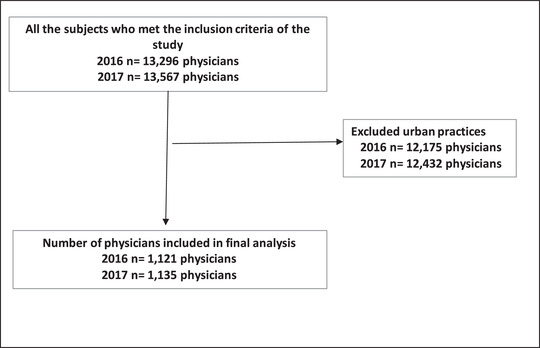
Inclusion/exclusion flowchart for 2016 and 2017 and rural physician cohorts

**FIGURE 2:**
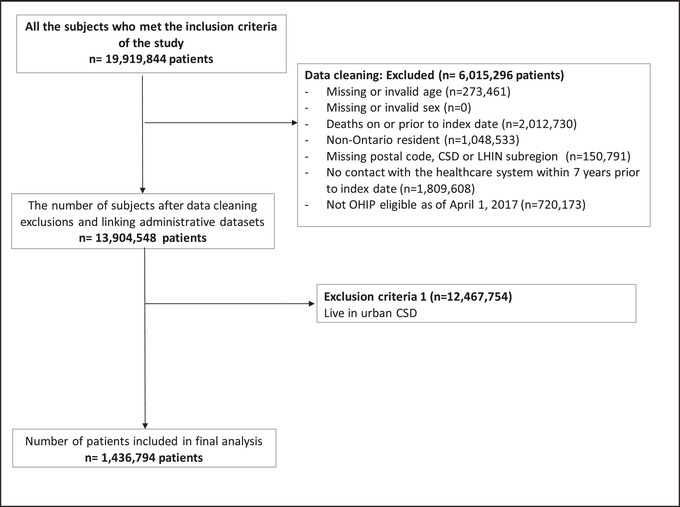
Inclusion/exclusion flow chart for patient cohort

After applying inclusion and exclusion criteria (Figure 1), the physician cohorts consisted of 1,121 and 1,135 physicians who worked in rural communities in Ontario in 2016 and 2017, respectively (comprising 8.4% of the Ontario physician workforce in each year). These cohorts were used to derive the physician retention measure. The study sample consisted of 1,436,794 rural residents (7.21% of the 2017 provincial population) (Figure 2). The majority of the study sample were under 60 years of age, male, had no comorbid conditions, and belonged to an FHT (Table 1). The sample had a mean of 3.79 primary care visits in 2017. There were 93,752 (6.53%) hospitalizations; 8,691 (0.6%) for ACSC.

**TABLE 1:** Characteristics of the study sample, all ages

Patient characteristics	
**Age at index date**	
<18	256,517 (17.85%)
18‐39	349,207 (24.30%)
40‐59	393,788 (27.41%)
60‐74	305,295 (21.25%)
74+	131,987 (9.19%)
Female, N (%)	713,240 (49.64%)
Number of comorbidities	
0	831,354 (57.86%)
1	377,141 (26.25%)
2	148,051 (10.30%)
3+	80,248 (5.59%)
**Primary care organization**	
CAP–Capitation, N (%)	311,202 (21.66%)
FFS–Enhanced fee for service, N (%)	153,782 (10.70%)
FHT–Family health team, N (%)	725,258 (50.48%)
NOG–Physician not in PEM, N (%)	57,038 (3.97%)
OGP–Other enrollment group, N (%)	29,962 (2.09%)
NOP–No physician, N (%)	159,552 (11.10%)
**Community characteristics**	
Physicians per 1,000 population, Mean (SD)	0.05 (0.26)
Community‐level physician retention	
No doctor	375,380 (26.13%)
Low retention	329,393 (22.93%)
Medium retention	397,401 (27.66%)
High retention	334,620 (23.29%)
**Health services utilization**	
Primary care visits, Mean (SD)	3.79 (7.50)
Specialist visits, Mean (SD)	2.59 (7.40)
Number of ED visits, Mean (SD)	0.70 (1.55)
**Outcomes**	
Hospitalization (all cause), N (%)	93,752 (6.53)
Hospitalization (ACSC), N (%)	8,691 (0.6)

Abbreviations: ACSC, ambulatory care‐sensitive condition; COPD, chronic obstructive pulmonary disease; ED, emergency department; HIV, human immunodeficiency virus; PEM, patient enrollment model.

Table 2 compares patient characteristics by community‐level physician retention. Given the large sample size, even relatively small differences between groups are statistically significant, so we focus on larger differences (eg, 4% or more) to highlight more meaningful differences between groups. No doctor communities have a smaller proportion of patients who belong to FHTs (45.12%) than the other communities, where more than 50% of the patients belong to FHTs. No doctor and high‐retention communities have lower event rates for hospitalization and ACSC hospitalization than low‐ and medium‐retention communities in the main analysis and sensitivity analyses (Table 3).

**TABLE 2:** Bivariate analysis of physician retention and patient, community, and health service utilization characteristics, all ages

	No Doctor (N = 375,380)	Low Retention (N = 329,393)	Medium Retention (N = 397,401)	High Retention (N = 334,620)	*P* value
**Patient characteristics**					
Age at index date					
<18	66,749 (17.78%)	58,584 (17.79%)	65,365 (16.45%)	65,819 (19.67%)	<.0001
18‐39	89,179 (23.76%)	80,160 (24.34%)	93,775 (23.60%)	86,093 (25.73%)	
40‐59	106,274 (28.31%)	88,701 (26.93%)	108,008 (27.18%)	90,805 (27.14%)	
60‐74	82,618 (22.01%)	71,366 (21.67%)	87,156 (21.93%)	64,155 (19.17%)	
74+	30,560 (8.14%)	30,582 (9.28%)	43,097 (10.84%)	27,748 (8.29%)	
Female, N (%)	182,338 (48.57%)	163,878 (49.75%)	200,430 (50.44%)	166,594 (49.79%)	<.0001
Number of comorbidities					
0	218,279 (58.15%)	189,541 (57.54%)	222,581 (56.01%)	200,953 (60.05%)	<.0001
1	99,043 (26.38%)	85,995 (26.11%)	107,438 (27.04%)	84,665 (25.30%)	
2	38,266 (10.19%)	34,610 (10.51%)	43,342 (10.91%)	31,833 (9.51%)	
3+	19,792 (5.27%)	19,247 (5.84%)	24,040 (6.05%)	17,169 (5.13%)	
**Primary care organization**					
CAP–Capitation, N (%)	79,404 (21.15%)	59,586 (18.09%)	97,294 (24.48%)	74,918 (22.39%)	<.0001
FFS–Enhanced fee for service, N (%)	42,857 (11.42%)	32,291 (9.80%)	42,138 (10.60%)	36,496 (10.91%)	
FHT–Family health team, N (%)	169,358 (45.12%)	174,038 (52.84%)	208,614 (52.49%)	173,248 (51.77%)	
NOG–Physician not in PEM, N (%)	17,165 (4.57%)	15,320 (4.65%)	9,983 (2.51%)	14,570 (4.35%)	
OGP–Other enrollment group, N (%)	14,901 (3.97%)	8,040 (2.44%)	4,040 (1.02%)	2,981 (0.89%)	
NOP–No physician, N (%)	51,695 (13.77%)	40,118 (12.18%)	35,332 (8.89%)	32,407 (9.68%)	
**Community characteristics**					
Physicians per 1,000 population, Mean (SD)	0.05 (0.26)	1.16 (1.10)	1.26 (0.75)	0.71 (0.59)	<.0001
**Health services utilization**					
Primary care visits, Mean (SD)	3.79 (7.50)	4.05 (7.66)	4.27 (7.42)	3.98 (7.47)	<.0001
Specialist visits, Mean (SD)	2.59 (7.40)	2.61 (7.54)	2.72 (7.70)	2.46 (7.27)	<.0001
Number of ED visits, Mean (SD)	0.70 (1.55)	0.77 (1.74)	0.70 (1.57)	0.65 (1.41)	<.0001

Abbreviations: ACSC, ambulatory care‐sensitive condition; COPD, chronic obstructive pulmonary disease; ED, emergency department; HIV, human immunodeficiency virus; PEM, patient enrollment model.

**TABLE 3:** Descriptive statistics of outcomes

	No doctors (N = 375,380)		Low retention (N = 329,393)		Medium retention (N = 397,401)		High retention (N = 334,620)	
	No. of events	Event rate (%)	No. of events	Event rate (%)	No. of events	Event rate (%)	No. of events	Event rate (%)
**All ages**								
Hospitalization (all cause)	24,467	6.52	22,381	6.79	26,055	6.56	20,849	6.23
Hospitalization (ACSC)	2,221	0.59	2,123	0.64	2,545	0.64	1,802	0.54
**Non‐northern, all ages**								
Hospitalization (all cause)	23,441	6.44	21,172	6.73	26,055	6.56	20,628	6.21
Hospitalization (ACSC)	2,141	0.59	2,055	0.65	2,545	0.64	1,790	0.54
**18‐74**								
Hospitalization (all cause)	17,174	6.18	15,264	6.35	16,563	5.73	14,324	5.94
Hospitalization (ACSC)	1,158	0.42	1,066	0.44	1,193	0.41	913	0.38

Abbreviation: ACSC, ambulatory care‐sensitive condition.

When investigating the effect of physician retention on the likelihood of all‐cause hospitalizations, we found that after controlling for other significant predictors (ie, number of comorbidities, primary care organization, and age‐sex interaction), compared to residents in low‐retention communities, residents of medium‐ and high‐retention communities were 0.888 (p = <.001) and 0.937 (p = <.001) times as likely to be hospitalized in 2017 (ie, a 11.2% and 6.3% reduction in hospitalization, respectively) (Table 4). Compared to patients with no comorbidities, patients with 1‐3 or more comorbidities were 1.163 (p = <.001), 1.371 (p = <.001), and 1.946 (p = <.001) times, respectively, more likely to be hospitalized in 2017 (ie, a 16.3%, 37.1%, and 84.6% increase in hospitalization, respectively). Compared to residents who belonged to FHTs, residents who belonged to CAP, FFS, and NOG‐type primary care organizations were less likely to be hospitalized in 2017 (0.842 [p = <.001], 0.780 [p = <.001], and 0.942 [p = .005], respectively; ie, a 15.8%, 22.0%, and 5.8% reduction in hospitalization, respectively), while residents who belonged to OGP or NOP organizations were more likely to be hospitalized (1.083 [p = .004] and 1.114 [p = <.001], respectively; ie, an 8.3% and 11.4% increase in hospitalization, respectively). Patients with larger numbers of primary care and specialist visits were more likely to be hospitalized, with every additional primary care and specialist visit increasing the likelihood of hospitalization by 1.155 (p = <.001) and 1.199 (p = <.001), respectively (ie, a 15.5% and 19.9% increase in hospitalization, respectively). Compared to females aged 18‐39 years, females in all other age groups were less likely to be hospitalized in 2017. In contrast, compared to males aged 18‐39 years, males of all other age groups were more likely to be hospitalized in 2017. In sensitivity analyses with the sample restricted to non‐northern rural communities, similar results were obtained for all‐cause hospitalization (Table 5).

**TABLE 4:** Adjusted odds ratio of all cause and ACSC hospitalization and 95% confidence intervals, all ages

	Hospitalizations				ACSC hospitalizations (all ages)			
	Adjusted odds ratio (95% CI)				Adjusted odds ratio (95% CI)			
	OR	Lower CL	Upper CL	*P* value	OR	Lower CL	Upper CL	*P* value
**Physician retention**								
No doctors	0.992	0.969	1.015	.499	1.005	0.943	1.071	.881
Low	REF	REF	REF		REF	REF	REF	
Medium	0.888	0.868	0.909	**<.001**	0.949	0.892	1.009	.095
High	0.937	0.915	0.960	**<.001**	0.918	0.858	0.981	**.012**
**Number of comorbidities**								
0	REF	REF	REF		REF	REF	REF	
1	1.163	1.138	1.189	**<.001**	10.831	9.504	12.342	**<.001**
2	1.373	1.335	1.412	**<.001**	32.061	27.853	36.904	**<.001**
3+	1.946	1.887	2.007	**<.001**	110.328	96.030	126.755	**<.001**
**Primary care organization**								
CAP–Capitation	0.842	0.825	0.861	**<.001**	0.834	0.787	0.884	**<.001**
FFS–Enhanced fee for service (CCM and FHG)	0.780	0.758	0.802	**<.001**	0.731	0.677	0.790	**<.001**
FHT–Family health team	REF	REF	REF		REF	REF	REF	
NOG–Physician not in PEM	0.942	0.904	0.982	**.005**	0.907	0.811	1.014	.086
OGP–Other enrollment group	1.083	1.026	1.143	**.004**	1.015	0.883	1.165	.837
NOP–No physicians	1.114	1.080	1.150	**<.001**	1.288	1.176	1.410	**<.001**
Primary care visits	1.055	1.054	1.056	**<.001**	1.035	1.034	1.036	**<.001**
Specialist visits	1.199	1.197	1.200	**<.001**	1.021	1.020	1.022	**<.001**
**Interactions between age and sex**								
Age among females								
<18	0.296	0.282	0.310	**<.001**	3.509	2.824	4.360	**<.001**
18‐39	REF	REF	REF		REF	REF	REF	
40‐59	0.284	0.275	0.294	**<.001**	0.827	0.689	0.992	.041
60‐74	0.346	0.335	0.357	**<.001**	1.310	1.106	1.552	**.002**
75+	0.672	0.650	0.695	**<.001**	2.033	1.719	2.404	**<.001**
Age among males								
<18	1.762	1.660	1.870	**<.001**	3.277	2.707	3.966	**<.001**
18‐39	REF	REF	REF		REF	REF	REF	
40‐59	1.742	1.656	1.833	**<.001**	0.941	0.796	1.113	.481
60‐74	2.441	2.323	2.566	**<.001**	1.176	1.003	1.378	.046
75+	3.580	3.397	3.773	**<.001**	1.716	1.463	2.013	**<.001**

Abbreviations: ACSC, ambulatory care‐sensitive condition; OR, odds ratio; PEM, patient enrollment model.

**TABLE 5:** Adjusted odds ratio of all cause and ACSC hospitalization and 95% confidence intervals, all ages in non‐northern communities

	Hospitalizations				ACSC hospitalizations (all ages)			
	Adjusted odds ratio (95% CI)				Adjusted odds ratio (95% CI)			
	OR	Lower CL	Upper CL	*P* value	OR	Lower CL	Upper CL	*P* value
**Physician retention**								
No doctors	0.992	0.969	1.016	.529	0.986	0.924	1.052	.673
Low	REF	REF	REF		REF	REF	REF	
Medium	0.912	0.891	0.934	**<.001**	0.947	0.890	1.007	.085
High	0.961	0.938	0.986	**.002**	0.916	0.856	0.980	**.011**
**Number of comorbidities**								
0	REF	REF	REF		REF	REF	REF	
1	1.170	1.144	1.197	**<.001**	10.646	9.323	12.157	**<.001**
2	1.372	1.333	1.411	**<.001**	31.432	27.256	36.247	**<.001**
3+	1.952	1.893	2.014	**<.001**	107.434	93.340	123.656	**<.001**
**Primary care organization**								
CAP–Capitation	0.840	0.822	0.858	**<.001**	0.836	0.788	0.886	**<.001**
FFS–Enhanced fee for service (CCM and FHG)	0.768	0.747	0.790	**<.001**	0.736	0.681	0.794	**<.001**
FHT–Family health team	REF	REF	REF		REF	REF	REF	
NOG–Physician not in PEM	0.730	0.696	0.766	**<.001**	0.811	0.716	0.920	**.001**
OGP–Other enrollment group	1.069	1.009	1.132	**.023**	1.043	0.903	1.205	.569
NOP–No physicians	1.069	1.034	1.104	**<.001**	1.285	1.172	1.409	**<.001**
Primary care visits	1.055	1.054	1.056	**<.001**	1.036	1.034	1.037	**<.001**
Specialist visits	1.199	1.197	1.200	**<.001**	1.021	1.020	1.022	**<.001**
**Interactions between age and sex**								
Age among females								
<18	0.290	0.277	0.304	**<.001**	3.652	2.923	4.563	**<.001**
18‐39	REF	REF	REF		REF	REF	REF	
40‐59	0.289	0.280	0.299	**<.001**	0.858	0.712	1.034	.108
60‐74	0.353	0.342	0.364	**<.001**	1.372	1.152	1.633	**<.001**
75+	0.687	0.664	0.711	**<.001**	2.116	1.781	2.515	**<.001**
Age among males								
<18	1.798	1.691	1.913	**<.001**	3.216	2.646	3.909	**<.001**
18‐39	REF	REF	REF		REF	REF	REF	
40‐59	1.808	1.716	1.906	**<.001**	0.938	0.791	1.112	.461
60‐74	2.545	2.419	2.679	**<.001**	1.191	1.014	1.400	**.034**
75+	3.724	3.529	3.930	**<.001**	1.723	1.465	2.026	**<.001**

Abbreviations: ACSC, ambulatory care‐sensitive condition; OR, odds ratio; PEM, patient enrollment model.

When examining the effect of physician retention on the likelihood of ACSC hospitalizations, after controlling for other significant predictors (ie, number of comorbidities, primary care organization, and age‐sex interaction), compared to residents in low‐retention communities, residents of high‐retention communities were 0.918 (p = .012) times as likely to be hospitalized for an ACSC in 2017 (ie, an 8.2% reduction in hospitalization for ACSC, respectively) (Table 4). Compared to those with no comorbidities, patients with 1‐3 or more comorbidities were 10.831 (p = <.001), 32.061 (p = <.001), and 110.328 (p = <.001) times, respectively, more likely to have an ACSC hospitalization in 2017 (ie, a 1,083.1%, 3,206.1%, and 11,032.8% increase in hospitalization for ACSC, respectively). Compared to residents who belonged to FHTs, residents who belonged to CAP and FFS‐type primary care organizations were less likely to be hospitalized for an ACSC in 2017 (0.834 [p = <.001] and 0.731 [p = <.001], respectively) (ie, a 16.6% and 26.9% reduction in hospitalization for ACSC, respectively), while NOP residents were 1.288 (p = <.001) times more likely to be hospitalized for an ACSC (ie, a 28.8% increase in hospitalization for ACSC). Patients with larger numbers of primary care and specialist visits were more likely to be hospitalized for ACSC, with every additional primary care and specialist visit increasing the likelihood of ACSC hospitalization by 1.035 (p = <.001) and 1.021 (p = <.001), respectively (ie, a 3.5% and 2.1% increase in hospitalization for ACSC, respectively). Compared to females aged 18‐39 years, females under 18, 60‐74, and 75+ were more likely to be hospitalized for ACSC in 2017. Similarly, compared to males aged 18‐39 years, males under 18, 60‐74, and 75+ were more likely to be hospitalized for ACSC in 2017. In sensitivity analyses with the sample restricted to non‐northern rural communities, or ages 18‐74 in all rural communities, similar results were obtained (Tables 5 and 6). When the sample for ACSC hospitalization is restricted to those that are 18‐74 years old, community‐level physician retention was not a significant predictor of ACSC hospitalization.

**TABLE 6:** Adjusted odds ratio of all cause and ACSC hospitalization and 95% confidence intervals, 18‐74

	Hospitalizations				ACSC hospitalizations (all ages)			
	Adjusted odds ratio (95% CI)				Adjusted odds ratio (95% CI)			
	OR	Lower CL	Upper CL	*P* value	OR	Lower CL	Upper CL	*P* value
**Physician retention**								
No doctors	0.983	0.957	1.011	.232	0.983	0.900	1.073	.694
Low	REF	REF	REF		REF	REF	REF	
Medium	0.856	0.833	0.880	**<.001**	0.976	0.894	1.065	.582
High	0.920	0.894	0.947	**<.001**	0.957	0.872	1.050	.354
**Number of comorbidities**								
0	REF	REF	REF		REF	REF	REF	
1	1.129	1.102	1.156	**<.001**	6.900	5.926	8.034	**<.001**
2	1.349	1.307	1.394	**<.001**	19.723	16.864	23.066	**<.001**
3+	1.732	1.666	1.800	**<.001**	71.158	60.980	83.034	**<.001**
**Primary care organization**								
CAP–Capitation	0.849	0.828	0.871	**<.001**	0.834	0.768	0.905	**<.001**
FFS–Enhanced fee for service (CCM and FHG)	0.790	0.765	0.816	**<.001**	0.767	0.690	0.851	**<.001**
FHT–Family health team	REF	REF	REF		REF	REF	REF	
NOG–Physician not in PEM	0.958	0.913	1.005	.078	0.894	0.769	1.040	.147
OGP–Other enrollment group	1.098	1.030	1.171	**.004**	1.008	0.829	1.226	.938
NOP–No physicians	1.050	1.011	1.089	**.011**	1.496	1.326	1.688	**<.001**
Primary care visits	1.042	1.042	1.043	**<.001**	1.036	1.035	1.038	**<.001**
Specialist visits	1.205	1.203	1.207	**<.001**	1.020	1.019	1.022	**<.001**
**Interactions between age and sex**								
Age among females								
18‐39	REF	REF	REF		REF	REF	REF	
40‐59	0.289	0.280	0.298	**<.001**	0.901	0.750	1.083	.266
60‐74	0.355	0.344	0.367	**<.001**	1.456	1.225	1.730	**<.001**
Age among males								
18‐39	REF	REF	REF		REF	REF	REF	
40‐59	1.647	1.567	1.731	**<.001**	1.036	0.875	1.227	.683
60‐74	2.302	2.192	2.417	**<.001**	1.329	1.130	1.564	**.001**

Abbreviations: ACSC, ambulatory care‐sensitive condition; OR, odds ratio; PEM, patient enrollment model.

In all multivariate regressions, the likelihood of all‐cause hospitalization or ACSC hospitalization did not differ between residents of communities with no doctor and residents of communities with low retention.

## DISCUSSION

We found that community‐level physician retention is a significant predictor of all‐cause hospitalization and ACSC hospitalization in rural communities in Ontario. These results are consistent with findings from an earlier study in another Canadian province.^10^ In our analyses, hospitalization includes admissions for maternity care and likely contributes to the age‐sex interaction that found female residents between 18 and 39 years were more likely than any other female age group to be hospitalized. It is unlikely, however, that maternity‐related admissions account for the relationship between community‐level physician retention and hospitalization. When we excluded northern communities, which have higher birth rates than other rural communities, or limited the analysis of ACSC hospitalization, community‐level physician retention remained a predictor of hospitalization. As expected, in all multivariate analyses, the likelihood of hospitalization and ACSC hospitalization increased with comorbidities.

Contrary to our hypothesis, “no doctor” communities did not have poorer outcomes than low‐retention communities. In our study, it is important to note that the “no physician” label does not indicate that communities do not have any health care resources. In many small communities in Ontario that do not have physicians who reside in the community, community health nurses (advanced masters‐trained nurses), nurse practitioners, or other allied health professionals provide routine primary care services, with physicians from other communities visiting regularly to provide medical services.^34^, ^35^ This model of care is typical of many small and remote communities in Canada, especially in the Northern territories.^34^ While these communities still have access to physician services, they do not have the type of access associated with having a physician based in the community.

Although the recruitment and retention of physicians in rural communities remains a longstanding challenge, few studies have examined the impact of a stable physician workforce by measuring physician retention. We devised a measure of community‐level physician retention as an alternative to continuity of care measures that rely on physician billing data. We grouped retention level by tertiles, based on the number of communities in each group and expert opinion. Alternately, we could have based tertile cutoffs solely on retention value (ie, 0‐62, 63‐99, and 100) but the difference between groups (eg, 99% vs 100% retention) would not have had any real‐world meaning. Now that initial analysis has demonstrated the association between retention on hospitalization, further research, including correlating cutoffs for established measures of continuity with community‐level physician retention, is needed to establish more evidence‐based, policy relevant cutoffs for community‐level physician retention. In addition, further study, on a broader range of health outcomes, is needed to understand the impact of physician turnover on rural resident health and inform alternate policy approaches.

### Limitations

We used a 1‐year cross‐sectional design. While a cross‐sectional design ensures that the outcome (hospitalization) and exposure (physician retention) occur in the same time period, they produce correlational rather than causal associations. Moreover, physician retention metrics based on 1 year of data may not capture longer term trends in physician retention. Future studies should examine a longer exposure to generate more stable estimates of retention and avoid mis‐labeling a community. While physician retention is calculated at the community level, individual residents in a low‐retention community may enjoy high continuity of care if their own physician remains in the community. Further research is needed to disentangle the effect of continuity with a single provider from the effects of community‐level physician retention. Our study used administrative health data, which do not capture many confounding variables that may contribute to hospitalization. Moreover, administrative health data do not capture services provided by nurses or other health care providers. These data are needed to capture the full‐range of health services, especially those provided in “no‐doctor” communities.

## CONCLUSIONS

Community‐level physician retention is significantly associated with all cause and ACSC hospitalization in rural communities in Ontario. Rural communities with medium or high physician retention had better outcomes than communities with low retention or no doctors. This initial study shows that community‐level physician retention is a promising measure of continuity appropriate for use in rural communities. Further research examining a wider range of health outcomes and including measures of physician retention over longer periods of time is needed to provide more robust evidence to inform health service policy in rural communities.

## FUNDING SOURCES

This study was supported by ICES, which is funded by an annual grant from the Ontario Ministry of Health (MOH) and the Ministry of Long‐Term Care (MLTC). The study was completed at the ICES Western site, where core funding is provided by the Academic Medical Organization of Southwestern Ontario, the Schulich School of Medicine and Dentistry, Western University, and the Lawson Health Research Institute. This study also received funding from Western University. Parts of this material are based on data and information compiled and provided by: MOH, CIHI. The analyses, conclusions, opinions, and statements expressed herein are solely those of the authors and do not reflect those of the funding or data sources; no endorsement is intended or should be inferred. Additional support was provided to Michael Green through the Brian Hennen Chair in Family Medicine, Queen's University.

## DISCLOSURES

None to declare.
